# Epsilon glutathione transferases possess a unique class-conserved subunit interface motif that directly interacts with glutathione in the active site

**DOI:** 10.1042/BSR20150183

**Published:** 2015-11-26

**Authors:** Jantana Wongsantichon, Robert C. Robinson, Albert J. Ketterman

**Affiliations:** * Institute of Molecular and Cell Biology, A*STAR (Agency for Science, Technology and Research), Biopolis, Singapore 138673; † Department of Biochemistry, National University of Singapore, 8 Medical Drive, Singapore 117597; ‡ Institute of Molecular Biosciences, Mahidol University, Salaya Campus, Nakhon Pathom 73170, Thailand

**Keywords:** dimer interface, epsilon crystal structure, GSTE6, insect glutathione transferase, protein structure

## Abstract

Epsilon class glutathione transferases (GSTs) have been shown to contribute significantly to insecticide resistance. We report a new Epsilon class protein crystal structure from *Drosophila melanogaster* for the glutathione transferase DmGSTE6. The structure reveals a novel Epsilon clasp motif that is conserved across hundreds of millions of years of evolution of the insect Diptera order. This histidine-serine motif lies in the subunit interface and appears to contribute to quaternary stability as well as directly connecting the two glutathiones in the active sites of this dimeric enzyme.

## Abstract (precis)

Analysis of a new structure of an Epsilon class glutathione transferase from *Drosophila melanogaster* reveals a highly conserved motif that spans the dimeric subunit interface and connects the two active sites.

## INTRODUCTION

Glutathione transferases (GSTs) are a superfamily of proteins found in all cellular organisms, which usually exist as multiple isoforms [1,2]. This extensive family has been categorized into at least 13 classes. Four classes, Omega, Sigma, Theta and Zeta, are found in all or most metazoans. There are also classes that appear to be found only in specific organisms such as in plants, Phi and Tau [3], or in arthropods, Delta and Epsilon [4]. One general function of this superfamily is the involvement in detoxication reactions for both endogenous and xenobiotic compounds. Other specific physiological roles have been identified for individual enzymes; in mammals the Sigma GST has been shown to be prostaglandin D_2_ synthase [5]; and in Drosophila an Omega GST was found to be a key synthase enzyme in pteridine biosynthesis of red eye pigments [6].

In insects, the Delta and Epsilon class GSTs make major contributions to detoxication of insecticides, contributing to insecticide resistance [7,8]. Currently, there are seven Epsilon structures available; allelic proteins with different ligands from the mosquito malaria vector *Anopheles gambiae* [9,10], two allelic proteins from a second major mosquito malaria vector *Anopheles funestus* [11] and a structure from the housefly *Musca domestica* [12]. Therefore, additional structural data would increase our understanding of this enzyme class.

In the present study, we present a novel crystal structure of an Epsilon GST (DmGSTE6) from *Drosophila melanogaster*. DmGSTE6 has significantly high activity for 4-hydroxynonenal (HNE) [13]. HNE was first hypothesized to be a secondary cytotoxic end product of lipid peroxidation that contributes to the aetiology of degenerative disorders like Alzheimer's [14] and Parkinson's disease [15]. HNE is now known to also be a concentration dependent signalling molecule that modulates several pathways [16]. GSTs may therefore regulate the HNE modulation of these signalling pathways. The gene encoding DmGSTE6 is of interest as it originates from a template gene, which during the course of Drosophila evolution has generated several Epsilon genes in the Epsilon gene cluster [17]. Within the DmGSTE6 structure we have identified a motif that has been conserved across millions of years of evolution. This motif lies at the interface extending into each subunit and is similar to, but more complex than, the interface motif called the ‘clasp’ motif previously reported for Delta GSTs [18]. Residues from the Epsilon motif also reside in the active site and are likely to contribute to an electron-sharing network [19].

## MATERIALS AND METHODS

### Production and purification of DmGSTE6

The DNA encoding DmGSTE6 was engineered into a pET-21d(+) (Novagen) plasmid and transformed into *Escherichia coli* BL21 (DE3) cells. Overnight stationary-phase cultures in Luria–Bertani (LB) broth (8 ml) were used to inoculate 800 ml LB broth, both supplemented with 100 μg/ml ampicillin and 34 μg/ml chloramphenicol. The cells were allowed to grow for 3 h at 310 K and then were cooled to 291 K and induced with 0.1 mM β-D-1-thiogalactopyranoside (IPTG) and incubated overnight for 16 h. The cultures were harvested at 7000 × ***g*** for 10 min and the pellets were kept at 253 K until used. The induced culture pellet was suspended with 20 ml of PBS buffer (140 mM NaCl, 2.7 mM KCl, 10 mM Na_2_HPO_4_, 1.8 mM KH_2_PO_4_, pH 7.3) containing 400 μl of 100 mg/ml lysozyme, 10 mM DTT and 10 μl of 1.4 M β-mercaptoethanol by gentle vortex-mixing. The cell suspension was incubated on ice for 20 min and the crude cell lysate was obtained by sonication. The lysed cells were centrifuged at 10000 × ***g*** and 277 K for 30 min. The soluble DmGSTE6 in the supernatant fraction was purified using a GSTrap FF column. The 5 ml column was equilibrated with five column volumes (CV) of PBS buffer. The supernatant was applied to the column with a flow rate of 5 ml/min. The non-specific binding proteins were eluted with 10 CV of PBS buffer. The bound GST was eluted with five CV of elution buffer (10 mM GSH, 50 mM Tris–HCl, pH 8.0, 10 mM DTT).

### Crystallization

DmGSTE6 at 15–20 mg/ml was crystallized in the presence of 10 mM GSH under a condition containing 20% PEG3350 and 0.2 M sodium thiocyanate in a 1:1 ratio by sitting drop vapour diffusion at 288 K. Protein crystals appeared within 3 days.

### Data collection and processing

X-ray diffraction data were collected on beamline I03 at Diamond Light Source (DLS) with an X-ray wavelength of 0.976 Å (1 Å=0.1 nm), to a resolution of 1.72 Å. The crystal was oscillated in the beam by 0.1° per frame over a range of 360°. Data were processed and scaled using xia2 [20] and AIMLESS from the CCP4 suite [21]. Initial phases were obtained by molecular replacement with PHASER [22] using a GST Epsilon class from *A. gambiae* (PDB: 2IMI) as the search model. Model building was performed with COOT [23] and restrained refinement with REFMAC in CCP4 [21] and PHENIX [24]. The data collection and refinement statistics are shown in Table 1. Molecular graphics and analyses were performed with the UCSF Chimera package [25] and PyMol (The PyMol Molecular Graphic System, version 1.5.0.3 Schrödinger, LLC). Chimera is developed by the Resource for Biocomputing, Visualization, and Informatics at the University of California, San Francisco (supported by NIGMS P41-GM103311). The atomic coordinates of the crystal structure have been deposited into the Protein Data Bank (PDB access code 4YH2).

**Table 1:** Data collection and refinement statistics for GSTE6 *Highest resolution shell is shown in parenthesis.

Data collection
Space group	C2
Cell dimensions	
*a*, *b*, *c* (Å)	176.4, 58.9, 122.8
*α*, *β*, *γ* (°)	90.0, 128.2, 90.0
Resolution (Å)	96.4–1.72 (1.76–1.72)
*R*_merge_	0.09 (0.69)
*I*/σ*I*	12.3 (2.4)
Completeness (%)	99.8 (99.9)
Redundancy	6.6 (6.9)
Refinement	
Resolution (Å)	96.4–1.72 (1.74–1.72)
No. reflections	104998 (3266)
*R*_work/_*R*_free_	0.157 (0.272)/0.188 (0.357)
No. atoms	8099
Protein	7120
Water	979
B-factors (Å^2^)	
Protein	24.3
Water	34.6
RMSDs	
Bond lengths (Å)	0.006
Bond angles (°)	1.057
Ramachandran	
Favoured (%)	98.1
Allowed (%)	1.7
Outliers (%)	0.2

### Sequence alignment

Sequence alignment is done by Clustal X, version 2.0.10 [26] and displayed with GeneDoc (http://www.psc.edu/biomed/genedoc).

## RESULTS AND DISCUSSION

In the present study, we have determined the structure of DmGSTE6 to supplement the currently known Epsilon structures (Figure 1, Table 1). This structure adopts the typically highly conserved GST fold with two domains comprising a βαβαββα motif in the N-terminus and an all α-helical domain in the C-terminus.

**Figure 1:**
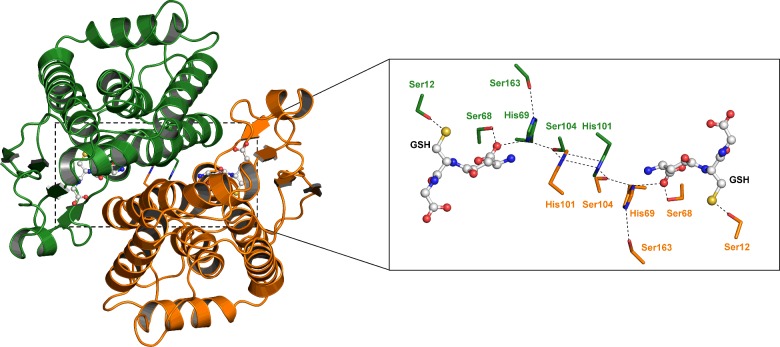
Ribbon representation of the *D. melanogaster* GSTE6 dimer Subunits (**A**) and (**B**) are distinguished by different colours, green and orange. The inset shows the ‘wafer’ histidine interface motif accompanied by serine residues connecting the two subunits from the active site of one subunit to the other. Ser^12^ is the catalytic residue.

Previously, we have carried out structure–function studies on Delta class GSTs, which identified an interface lock-and-key ‘clasp’ motif that is structurally conserved within the Delta class enzymes [18]. Amino acid sequence alignments predict that this motif may also be conserved in the Epsilon class GSTs. The structural motif appears to affect protein dynamics and therefore influences substrate specificity, enzyme activity and protein stability. Analysis of the clasp formation in this Epsilon DmGSTE6 reveals that the interface motif is more complex than observed for the Delta class. The Epsilon clasp motif comprises an extended ‘wafer’ arrangement of four histidines (two contributed from each subunit), which is supported by interactions with several conserved serines from helices 3, 4 and 6 (Figure 1). This elongated motif stretches across the interface deep into both subunits of the homodimer. His^101^ from one subunit wraps around the His^101^ from the other subunit to generate the clasp motif also seen in the Delta GST class [18]. This arrangement of the histidines involves aromatic ring stacking and pi–pi interaction of the two residues. In Delta class GSTs, the clasp motif has been shown to stabilize the quaternary structure as well as have a role in subunit communication between active sites [18]. The structural contributions of the clasp motif in the Delta GST also have an impact on catalytic specificity and the efficiency of the enzyme. In the Epsilon DmGSTE6, a second histidine in each subunit, His^69^, interacts with clasp His^101^ from the other subunit (Figure 1). These His–His interactions are also supported by three serines, Ser^68^, Ser^104^, Ser^163^, in each subunit (Figure 1). This extended motif would appear to play a structural role, as it is internal and spans the subunit interface. Furthermore, Ser^68^ directly interacts with GSH in the active site as the Ser^68^ OG and N atoms are within 2.5–3.3 Å of the GSH O11 and O12 atoms (Figure 1). The His^69^ ND1 atom is also within the interaction distance of 3.5 Å of the GSH O12 atom. Both Ser^68^ and His^69^ are located at the N-terminus of alpha helix 3 and possibly exploit the helix dipole to strengthen these interactions. Residues Ser^68^ and His^69^ in DmGSTE6 are the equivalent residues to Ser^65^ and Arg^66^, in the active site of the Delta class AdGSTD3, which are reported to be components of an electron-sharing network utilized for catalysis [19,27,28]. An amino acid sequence alignment of the six Epsilon GSTs, for which structures are available, is shown in Figure 2. The His wafer residues are conserved in these Epsilon class proteins and superposition of their structures shows that this sequence conservation translates into side chain position conservation in their 3D structures (Figure 3).

**Figure 2:**
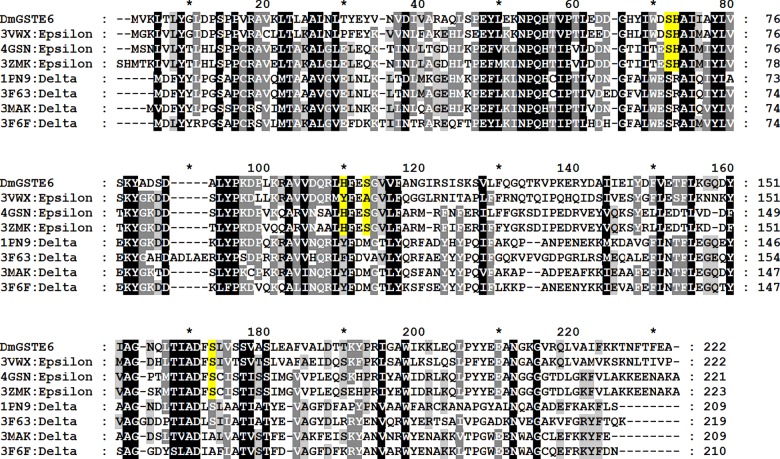
Amino acid alignment of the Epsilon GSTs compared with Delta GSTs with available crystal structures Highlighted in yellow is the conserved histidine wafer interface motif consisting of two histidines and three supporting serines from each subunit. DmGSTE6 in the present study refers to *D. melanogaster* GST Epsilon 6. The PDB ID codes are: 3VWX *M. domestica* Epsilon; 4GSN *A. gambiae* Epsilon 2; 3ZMK *A. funestus* Epsilon 2; 1PN9 *A. gambiae* Delta 1; 3F63 *Anopheles dirus* Delta 4; 3MAK *D. melanogaster* Delta 1; 3F6F *D. melanogaster* Delta 10.

**Figure 3:**
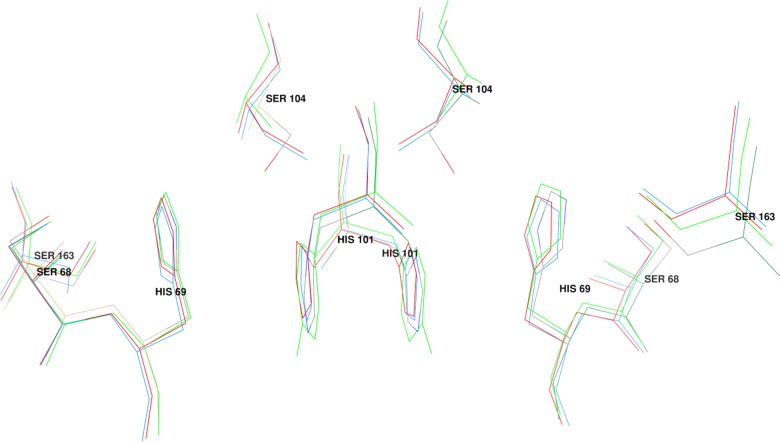
Superposition of the histidine interface motif of Epsilon GSTs from four species The residue numbering refers to the *D. melanogaster* GST Epsilon 6. The superimposed structures are 4YH2 (the present study, *D. melanogaster*), 2IMI (*A. gambiae*), 3ZML (*A. funestus*) and 3VWX (*M. domestica*).

The current structure also shows that the active site pocket for Epsilon class GSTs is more in a ‘closed’ configuration than that of Delta class. Major contributions to this difference arise from the arrangement of helices, especially the positioning of alpha helix 4 (Figures 4a and 4b). This offers an extra GSH interaction with Arg^113^ from the C-terminal domain, which is a novel feature and perhaps is unique to Epsilon class GSTs (Figures 4c and 4d). To validate the conservation of this arginine and other GSH-binding residues, all Epsilon and Delta class GSTs were retrieved from complete genomes of *D. melanogaster* and *A. gambiae* for protein sequence analysis. Based on previous annotation, this includes 14 Epsilon/12 Delta class GSTs from *D. melanogaster* [13] and 8 Epsilon/15 Delta class GSTs from *A. gambiae* [29]. Besides the highly conserved catalytic serine (Ser^12^), we categorized GSH-binding residues into three different groups namely the GSH glycyl moiety, the GSH glutamyl moiety and the C-terminal domain interactor (Figure 5). The impact of protein–ligand interactions toward the glycyl and glutamyl regions of GSH have been extensively studied for the Delta class GSTs. Amino acid conservation in these positions has been reported to play a significant role in enzyme catalysis, substrate specificity and structural integrity of the enzyme [19,27,28,30]. The histidine that interacts with the glycyl moiety of GSH (His^53^ in DmGSTE6) is highly conserved in both Delta and Epsilon classes of GSTs (Figure 5a). Amino acid replacement of the equivalent residue to His^53^ in a Delta class GST exhibits a catalytic efficiency approximately 5200-fold lower than observed for the wild-type [28]. For the GSH glutamyl moiety (Figure 5b), this region of interactions also represents functionally conserved residues in both GST classes, and influences protein folding, enzyme kinetics, the rate-limiting step, GSH ionization and the electron-sharing network in catalysis [19,27,30].). It is of interest that the strictly conserved His^69^ in Epsilon class, which is involved in GSH glutamyl binding, is also one of the ‘wafer’ histidines in the interface motif. This position has a significant role in GSH-binding affinity, substrate specificity and protein folding. As a result, His^69^, which links the subunits across the dimeric interface and communicates from one active site to the other, may be an important regulating residue for the Epsilon class of GSTs. The final pair of GSH-binding residues in Epsilon class GSTs lies in the C-terminal domain, Phe^106^ and Arg^113^. This region is of particular interest since it lies on alpha helix 4, which is positioned relatively closer to the N-terminal domain compared with the Delta class GSTs. Arg^113^ is conserved only in the Epsilon class (Figure 5c) and it interacts with the glutamyl part of GSH, suggesting that this residue may play a role in modulating GSH/hydrophobic substrate conjugation. Additionally, structural comparison focusing on alpha helix 4 also shows that Phe^108^ in GSTE6, though conserved in both insect-specific classes of GST (Figure 2), is close enough for hydrophobic interaction with GSH only in the Epsilon class. Distance from Phe^108^ CE1 atom of DmGSTE6 to GSH CB1 is 3.5 Å whereas the closest distance between dmGSTD1 and GSH is 5.6 Å from Tyr^106^ CE2 atom to GSH OE1 atom.

**Figure 4:**
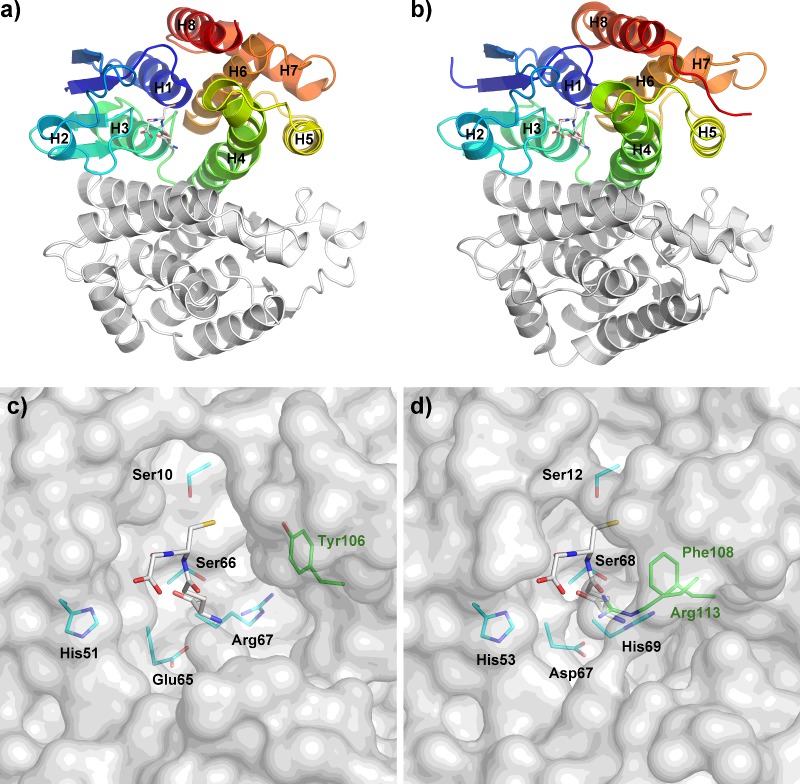
Active site pockets of DmGSTE6 in comparison with dmGSTD1 Arrangement of helices in dmGSTD1 (**a** and **c**) and DmGSTE6 (**b** and **d**) contributes to the shape of active site pockets. Stick representation of glutathione is in white and its interacting residues from the N-terminal and the C-terminal domain are in cyan and green, respectively. Tyr^106^ in panel **c** is for comparison only.

**Figure 5:**
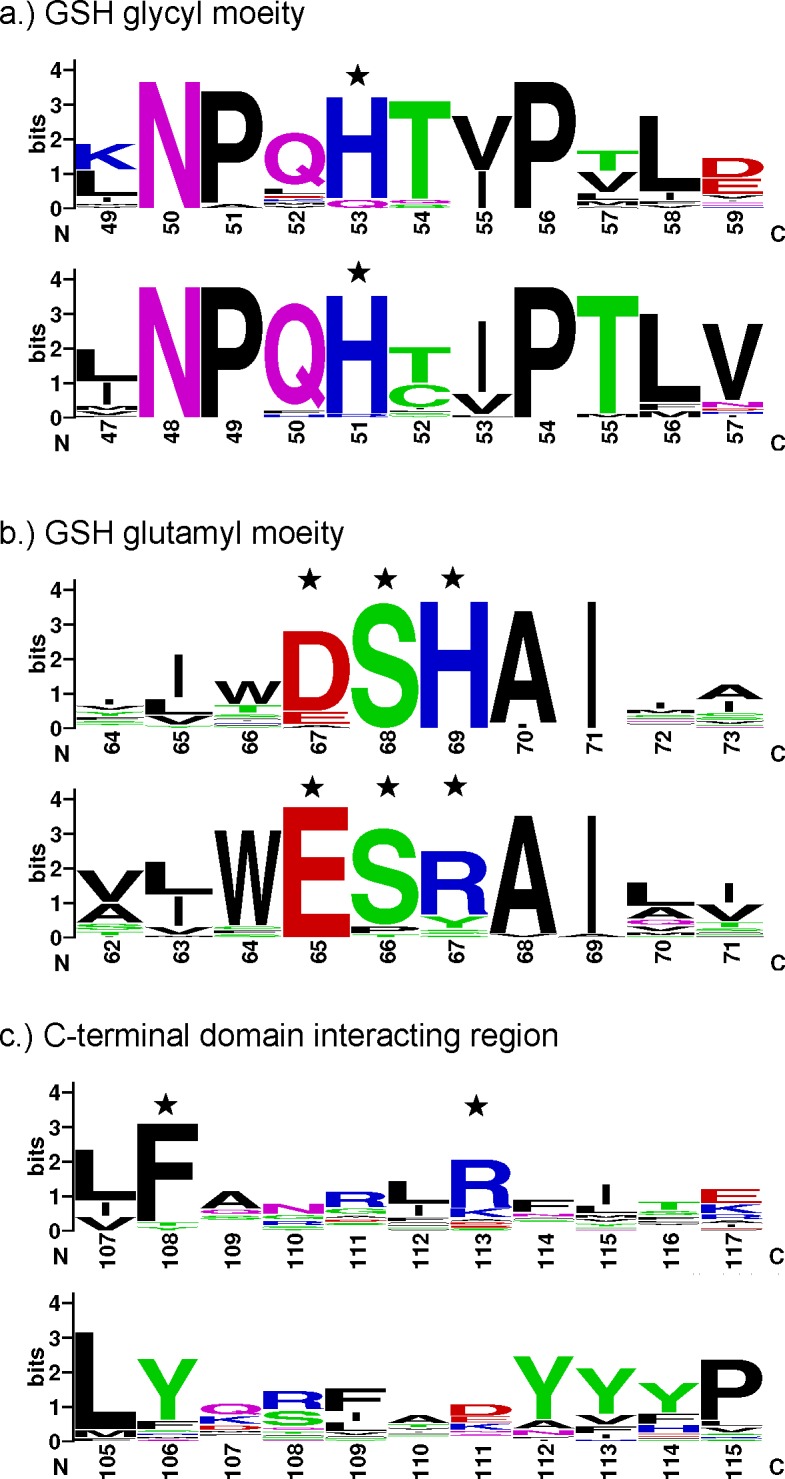
Graphical representation of amino acid multiple sequence alignment for glutathione binding residues of Epsilon and Delta class GSTs from *D. melanogaster* and *A. gambiae* genomes The glutathione interacting residues are indicated with stars. The top panel in each category represents Epsilon class GSTs and the bottom panel represents Delta class GSTs with respective residue numbers on an *X*-axis. The image was generated by WebLogo [44,45]. A logo represents each column of the alignment by a stack of letters, with the height of each letter proportional to the observed frequency of the corresponding amino acid, and the overall height of each stack proportional to the sequence conservation at that position [45]. The letters of each stack are ordered from most to least frequent, so that one may read the consensus sequence from the tops of the stacks [45]. Single letter amino acid symbols are coloured according to their chemical properties; polar amino acids (G, S, T, Y, C, Q, N) are green, basic (K, R, H) blue, acidic (D and E) red, and hydrophobic (A, V, L, I, P, W, F, M) amino acids are black [45].

As stated above, in *D. melanogaster* DmGSTE6 has significantly high activity for HNE although not as great as reported for human GSTA4 [31], it is greater than what has been observed for two other human Alpha GSTs, GSTA1 and GSTA2 [32]. Therefore superposition of the structure of human Alpha class GSTA4 with GSH–HNE conjugate analogue in the active site (PDB ID: 3IK7; [33]) and the DmGSTE6 allowed us to identify possible residues involved in HNE binding. In GSTA4 these residues have been identified as Tyr^9^ (catalytic residue); Arg^15^ (N-terminus of helix 1); Ile^107^, Met^108^, Phe^111^ (helix 4); Tyr^212^, Val^216^, Tyr^217^, Phe^220^ (helix 8) [33]. From superposition the residues for DmGSTE6 that would generate a hydrophobic binding cavity for HNE appear to be Ser^12^ (catalytic residue); Pro^11^, Pro^13^, Pro^14^ (N-terminus helix 1); Ile^36^ (coil between beta strand 2 and helix 2); Phe^108^, Ile^112^, Arg^113^, Leu^120^ (helix 4); Phe^174^ (C-terminus helix 7); Leu^209^, Phe^213^ (helix 9). Although appearing to generate a similar hydrophobic cavity the residues of DmGSTE6 originate from different regions of the protein. This suggests convergent evolutionary pressure occurred in the Epsilon and Alpha GST classes to independently obtain the HNE activity.

Both Delta and Epsilon class GSTs have been shown to be important for contributing to insecticide resistance in mosquito malaria and dengue vectors [9–11,34]. At present, both GST classes, Delta and Epsilon, appear to be found only in arthropods. Delta GSTs have been reported in the arthropod *Sarcoptes scabiei*, a parasitic arachnid [35], a copepod crustacean *Tigriopus japonicus* [36] and the Chinese mitten crab *Eriocheir sinensis* [37]. Genomes from the four major insect orders, Coleoptera (beetle), Diptera (true flies), Hymenoptera (wasps, ants and bees) and Lepidoptera (moths and butterflies), have been reported to possess Delta GSTs [38]. In contrast, the current fully sequenced Hymenoptera genomes (*Apis mellifera* and *Nasonia vitripennis*) lack the Epsilon class, which suggests that Epsilon class GSTs appeared after the Hymenoptera divergence, approximately 238.5–307.2 million years ago [39,40]. The currently available Epsilon structures are from species of the Diptera order which is thought to have rapidly radiated during the Triassic period, 201–252 million years ago [41–43]. As a frame of reference, it is estimated that humans and mice diverged 62–100 million years ago [39]. Thus the Epsilon GST class has been evolving for a significant period of time, while maintaining this distinct Epsilon clasp structural motif in species across the Diptera order. This suggests that the Epsilon clasp motif serves a critical function, possibly in structure maintenance and in contributing to catalysis through the electron-sharing network.

### Postscript

During the writing of this manuscript, two newly deposited structures of Drosophila GSTE6 (4PNF) and GSTE7 (4PNG) appeared in the PDB. This DmGSTE6 has structural similarity to the one in the present study (4YH2) with RMSD of 0.24 Å including 2840 atoms.

## Abbreviations

CV: column volumes
DmGSTE6: *Drosophila melanogaster* glutathione transferase epsilon 6
GST: glutathione transferase
HNE: 4-hydroxynonenal
LB: Luria–Bertani
